# Editorial: COVID 19: New Variants and Host Demography

**DOI:** 10.3389/fcimb.2022.905817

**Published:** 2022-05-02

**Authors:** Ginpreet Kaur, Hardeep Singh Tuli

**Affiliations:** ^1^ Shobhaben Pratapbhai Patel School of Pharmacy and Technology Management, Shri Vile Parle, Kelavani Mandal, Narsee Monjee Institute of Management Studies (SVKM’s NMIMS), Mumbai, India; ^2^ Department of Biotechnology, Maharishi Markandeshwar (Deemed to be University), Mullana, Ambala, India

**Keywords:** SARS-CoV-2, vaccination, demographic changes, physiological effects, treatment strategy

As the world continues to battle the SARS-Cov-2 virus, the mutants being sequenced suggest a grim chance of avoiding a very possible third wave. The mutation landscape of SARS-CoV-2 has been under constant global scrutiny to understand the effect of these changes on the infectivity and antigenicity of the virus. While most mutations are of little to no consequence, sometimes the virus acquires a mutation that gives it an advantage over other strains. Despite the virus’s sluggish mutation rate, researchers have catalogued more than 12,000 mutations in SARS-CoV-2 genomes. Compared with HIV, SARS-CoV-2 changes much more slowly as it spreads. The variants of concern have mostly been identified with a mutation in the gene encoding the spike protein, which helps virus particles to penetrate cells.

## New Variants

The D614G mutation took place at the 614th amino-acid position of the spike protein, the amino acid aspartate was regularly being replaced by glycine because of a copying fault that altered a single nucleotide in the virus’s 29,903-letter RNA code. All vaccines worked against this which is why the spread and infectivity was controlled. An interesting paper presented by Tuli et al. highlights the molecular evolution of SARS-CoV-2 variants, and effective targeting using vaccines. Mohammad et al. presented an article on the variation of structural proteins, corresponding to new genetic variants, and the adoption of a genomics-based approach to further our understanding of their effects. The 3 new variants that have rapidly become dominant within their respective countries have raised concerns include B.1.1.7 (also known as VOC-202012/01), 501Y.V2 (B.1.351), and P.1 (B.1.1.28.1).All three variants have the N501Y mutation, which changes the amino acid asparagine (N) to tyrosine (Y) at position 501 in the receptor-binding domain of the spike protein.

A new variant has recently been identified in West Bengal, India, popularly being called as ‘Triple Mutant Variant’ with the E484K, a major immune-escape mutation. The team of Bhat et al. highlighted the importance of mutation surveillance, using biological and computational models. The article by Singh et al. explores the emergence of new variants of SARS-CoV-2, and the effectiveness of vaccines in their management.

## Treatment Options for the Future

Variants are going to continue to mutate and at some point its going to happen that more of these will be able to evade the immune system. A few possible ways that are being discussed to mitigate the rate of mutation include a third dose of the same vaccine, a slightly modified booster dose and combinatorial dosing. The article by Rana et al. offers an in-depth insight into the future perspectives and possibilities for newer treatment strategies, moving forward. In addition to this, the article by Kushwaha et al. highlights the identification of natural inhibitors against SARS-CoV-2 and identification of druggable targets, using various approaches. Caldera-Crespo et al. presented an insightful paper on the various experimental models of COVID-19, while Singh et al. provided an enriching look into the future perspectives of COVID-19 management, using antibody-based therapy.

## New Variant Demography

After spending much of last year affecting elderly patients, healthcare workers are now seeing a demographic shift: young and middle-aged adults are becoming a growing share of the patients in COVID-19 hospital wards. In addition to the mapping of disease progression across different ages, this issue explores the impact of ethnicity on the incidence of the disease. The article by Al Zahmi et al. explains the difference of COVID-19 impact among various ethnicities across the globe. In addition to this, the article by Statsenko et al. highlights the impact of age and sex on the severity of COVID-19, based on evidence obtained from radiologic and clinical data. Nayak et al. aimed to provide an insight into the host response to existing and emerging variants of SARS-CoV-2, in patients with hepatic and gastrointestinal complications. With regards to analysis of the emergence of new variants in the Indian subcontinent, the papers presented by Rana et al. and Kandelwal et al. provided a thorough overview of the severity and extent of spreading of newer variants.

## Side Effects of Vaccines

The COVID-19 vaccines can cause mild adverse effects after the first or second dose, including pain, redness or swelling at the site of vaccine shot, fever, fatigue, headache, muscle pain, nausea, vomiting, itching, chills and joint pain, and can also rarely cause anaphylactic shock. The specific cause of the anaphylactic reactions remains unknown. The Moderna and Pfizer–BioNTech vaccines use hollow lipid nanoparticles, linked with PEG, which have been known to cause allergic reactions.

A larger body of studies are now showing an increased incidence of neurological manifestations among patients hospitalized with COVID-19. The complications include: acute encephalopathy, headaches, loss of sense of taste or smell, coma and strokes. An interesting paper submitted by Rustagi et al. maps the effects of COVID-19 vaccination in Asian countries, using machine learning. We hope that you all will enjoy the reading of this thematic issue on “Current Aspects in Chemopreventive Strategies” from Frontiers in Pharmacology.

## Author Contributions

All authors listed have made a substantial, direct, and intellectual contribution to the work and approved it for publication.

## Conflict of Interest

The authors declare that the research was conducted in the absence of any commercial or financial relationships that could be construed as a potential conflict of interest.

## Publisher’s Note

All claims expressed in this article are solely those of the authors and do not necessarily represent those of their affiliated organizations, or those of the publisher, the editors and the reviewers. Any product that may be evaluated in this article, or claim that may be made by its manufacturer, is not guaranteed or endorsed by the publisher.

